# *catena*-Poly[[[*trans*-diaqua­bis(pyridine-κ*N*)cobalt(II)]-μ-(4-{*N*′-[1-(3-acetyl-4-methyl-1*H*-pyrazol-5-yl)ethyl­idene]hydrazino}benzoato-κ^3^ *O*:*N*,*N*′)-[bis­(pyridine-κ*N*)cobalt(III)]-μ-(4-{*N*′-[1-(3-acetyl-4-methyl-1*H*-pyrazol-5-yl)ethyl­idene]hydrazino}benzoato-κ^3^ *N*,*N*′:*O*)]perchlorate 3.66-hydrate]

**DOI:** 10.1107/S1600536808002675

**Published:** 2008-01-30

**Authors:** Larysa Penkova, Mikhail P. Azarkh, Matti Haukka, Franc Meyer, Igor O. Fritsky

**Affiliations:** a National Taras Shevchenko University, Department of Chemistry, Volodymyrska str. 64, 01033 Kiev, Ukraine; b University of Joensuu, Department of Chemistry, PO Box 111, FI-80101 Joensuu, Finland; c Institut für Anorganische Chemie, Universität Göttingen, Tammannstrasse 4, 37077 Göttingen, Germany

## Abstract

The title compound, {[Co_2_(C_15_H_14_N_4_O_3_)_2_(C_5_H_5_N)_4_(H_2_O)_2_]ClO_4_·3.66H_2_O}_*n*_, is a one-dimensional coordination polymer, with both Co^II^ and Co^III^ centres in its structure. The ligand environment surrounding Co^III^ is formed by two *N*,*N*-chelating pyrazole-containing ligands and two pyridine mol­ecules in axial positions. The high-spin Co^II^ ions, situated at crystallographic centres of inversion, exhibit a distorted octa­hedral coordination mode. The ClO_4_^−^ anion is linked to the polymer chain *via* hydrogen bonds. The chains are connected by hydrogen bonds to produce a three-dimensional structure.

## Related literature

For related literature, see: Dalai *et al.* (2002 ▶); Eisenwiener *et al.* (2007 ▶); James (2003 ▶); Li & Xiao (2004 ▶); Min *et al.* (2002 ▶); Mukherjee (2000 ▶); Sato *et al.* (1999 ▶); Takahashi *et al.* (2006 ▶); Xiao *et al.* (2005 ▶); Yin *et al.* (2007 ▶); Zhu *et al.* (2004 ▶).
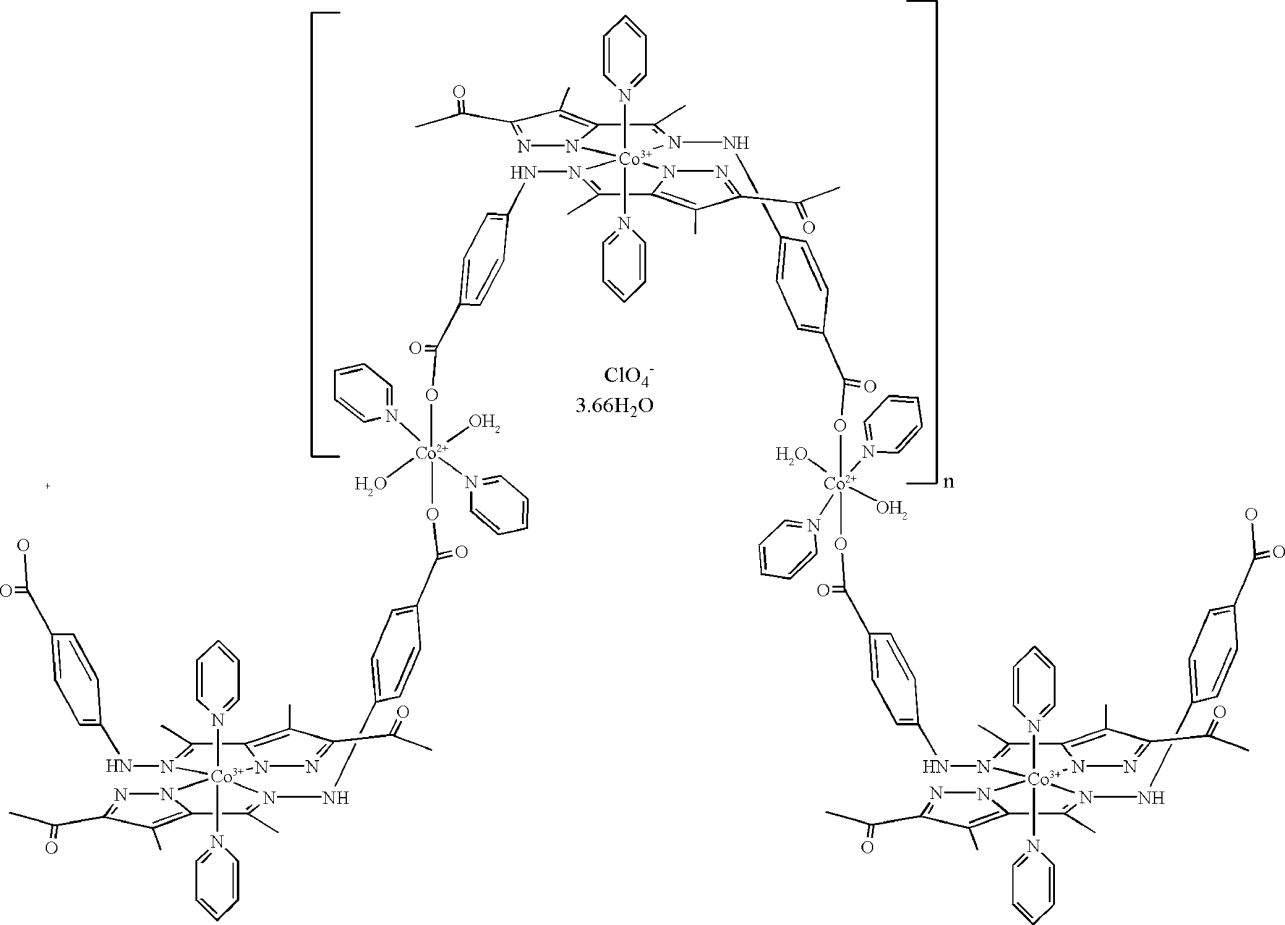


## Experimental

### 

#### Crystal data

[Co_2_(C_15_H_14_N_4_O_3_)_2_(C_5_H_5_N)_4_(H_2_O)_2_]ClO_4_·3.66H_2_O
*M* *_r_* = 1232.38
Triclinic, 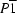

*a* = 10.1128 (2) Å
*b* = 13.9615 (4) Å
*c* = 20.1840 (6) Å
α = 85.969 (2)°
β = 80.844 (2)°
γ = 84.660 (2)°
*V* = 2796.79 (13) Å^3^
*Z* = 2
Mo *K*α radiation
μ = 0.72 mm^−1^
*T* = 120 (2) K
0.22 × 0.14 × 0.06 mm

#### Data collection

Nonius KappaCCD diffractometer
Absorption correction: multi-scan (*SORTAV*; Blessing, 1995 ▶) *T*_min_ = 0.866, *T*_max_ = 0.959
37217 measured reflections
9843 independent reflections
7347 reflections with *I* > 2σ(*I*)
*R*_int_ = 0.058

#### Refinement

*R*[*F* ^2^ > 2σ(*F* ^2^)] = 0.046
*wR*(*F* ^2^) = 0.125
*S* = 1.04
9843 reflections
763 parameters
H-atom parameters constrained
Δρ_max_ = 0.89 e Å^−3^
Δρ_min_ = −0.89 e Å^−3^

### 

Data collection: *COLLECT* (Bruker, 2004 ▶); cell refinement: *DENZO*/*SCALEPACK* (Otwinowski & Minor, 1997 ▶); data reduction: *DENZO*/*SCALEPACK*; program(s) used to solve structure: *SIR2004* (Burla *et al.*, 2005 ▶); program(s) used to refine structure: *SHELXL97* (Sheldrick, 2008 ▶); molecular graphics: *DIAMOND* (Brandenburg, 2006 ▶); software used to prepare material for publication: *SHELXL97*.

## Supplementary Material

Crystal structure: contains datablocks global, I. DOI: 10.1107/S1600536808002675/im2050sup1.cif

Structure factors: contains datablocks I. DOI: 10.1107/S1600536808002675/im2050Isup2.hkl

Additional supplementary materials:  crystallographic information; 3D view; checkCIF report

## Figures and Tables

**Table 1:** Hydrogen-bond geometry (Å, °)

*D*—H⋯*A*	*D*—H	H⋯*A*	*D*⋯*A*	*D*—H⋯*A*
O1—H1*O*⋯O3	0.96	1.76	2.677 (3)	159
O1—H1*P*⋯O10	0.91	2.02	2.903 (4)	166
O1—H1*P*⋯Cl1	0.91	2.91	3.795 (2)	166
O8—H8*O*⋯O13^i^	0.95	1.86	2.789 (4)	166
O8—H8*P*⋯O6^ii^	0.93	1.84	2.734 (4)	161
O13—H13*O*⋯O4	0.91	2.01	2.909 (4)	167
O13—H13*P*⋯O14^iii^	0.93	1.94	2.835 (5)	160
O14—H14*O*⋯O15	0.95	1.86	2.809 (7)	178
O14—H14*P*⋯O6	0.96	1.83	2.751 (5)	162
N2—H2*N*⋯N8	0.95	2.03	2.924 (4)	155
N2—H2*N*⋯N9	0.95	2.54	3.173 (4)	124
N11—H11*N*⋯N5	0.97	1.98	2.864 (4)	151
N11—H11*N*⋯N4	0.97	2.49	3.092 (4)	120

Symmetry codes: (i) 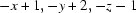; (ii) 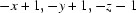; (iii) 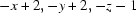.

**Fig. 1.**
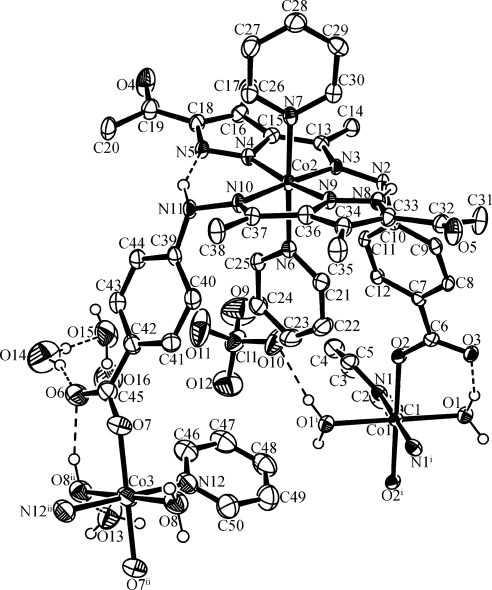
Monomeric fragment in the crystal structure of (I) with displacement ellipsoids drawn at the 50% probability level. Disordered positions of the water molecules (O15 and O16) and H-atoms not participating in hydrogen bonds are omitted for clarity. Symmetry code: i=1 - x, 1 - y, -z; ii=1 - x, 1 - y, -1 - z.

**Fig. 2.**
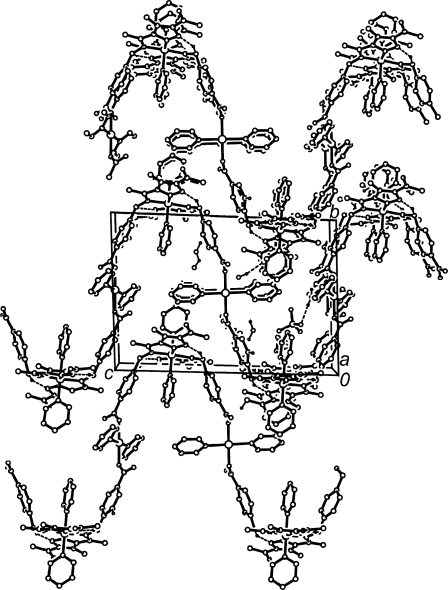
Crystal packing of (I). Disordered positions of the water molecules (O15 and O16) and H-atom not participating in hydrogen bonds are omitted for clarity.

## supplementary crystallographic information

### Comment

Pyrazole-based chelating ligands form a variety of coordination complexes providing various coordination geometries and nuclearities (Mukherjee, 2000; Eisenwiener *et al.*, 2007). They are also capable to form coordination polymers which are of great interest from the point of view of material chemistry and condensed matter physics (Dalai *et al.*, 2002; James, 2003; Min *et al.*, 2002; Takahashi *et al.*, 2006; Yin *et al.*, 2007). As it was previously reported (Li & Xiao 2004; Xiao *et al.*, 2005), rigid bridging ligands such as terephthalate anion and its derivatives are good precursors for zigzag coordination polymers. From this point of view, pyrazole-derived ligands with additional donor groups attached to the 3 and 5 positions of the heterocycle are promising precursors for metal-containing chains and frameworks. The preparation and crystal structure of (I), a novel one-dimensional coordination polymer on the basis of the 4-{*N*'-[1-(5-acetyl-4-methyl-2*H*-pyrazol-3-yl)-ethylidene]-hydrazino} benzoic acid (*L*) incorporating the alternating Co^II^ and Co^III^ ions, is reported herein.

L coordinates the Co^III ^ion *via* two nitrogen atoms of the 1*H*-pyrazole and hydrazone moieties (Fig.1). Two *trans*-disposed ligands form five-membered chelates in the equatorial plane. Co—N bond distances are in the range of 1.875 (2) – 1.949 (3) Å and are close to those in the [(2*S*,3S)-α-Me—*N*,*N*'-bis(salicylidene)butane- 2,3-diaminato]bis(pyridine)-cobalt(III) reported by Sato *et al.*, 1999 [average Co—N = 1.911 (5) Å]. The axial positions are occupied by the pyridine molecules [Co2—N7 = 1.978 (3) Å, Co2—N6 = 1.984 (3) Å]. The Co^III^ ion is therefore situated in a distorted octahedral environment. Bond angles at Co^III^ ranged from 80.6 (1)° to 100.0 (1)°.

Coordination polyhedra of both Co1 and Co3 ions display distorted compressed octahedra. At both metal ions two pyridine and two water molecules are equatorially coordinated in *trans*-positions. The Co—N(py) and Co—O(H_2_O) distances are in good agreement with those in diaqua-diformato-dipyridine-cobalt(II) (Zhu *et al.*, 2004), where they are equal to 2.159 (4) and 2.143 (3) Å, respectively. The axial contacts between Co^II^ ions and coordinated carboxylic groups of *L* are somewhat shorter than those in the equatorial plane [Co1—O2 = 2.040 (2) Å, Co3—O7 = 1.988 (2) Å].

The one-dimensional-polymeric chain displays a zigzag conformation due to nonplanarity of the pyrazole-containing ligand which binds both Co^III ^and Co^II^ ions. The intermetallic separations are 9.606 (1) Å for Co1···Co2 and 9.770 (1) for Co2···Co3.

A part of the crystal packing of (I) is presented in Fig. 2. The polymeric chains are spread along *c* axis. The translational chains are connected through hydrogen bonds [O8—H···O13^i^, O8—H···O(6)^ii^ and O13)—H···O14^iii^] (Table 1) in a three-dimensional structure.

### Experimental

To the solution of the ligand (0.0434 g, 0.1 mmol) in methanol (5 ml) the aqueous solution of Co(ClO_4_)_2_ (0.1 *M*, 2 ml) was added. The mixture was stirred for 10 min and then vapours of pyridine were diffused into the solution. The reaction mixture produced a brown precipitate that was filtered off. The remaining red solution was diluted with methanol (*ca* 5 ml) and exposed to slow diffusion of diethyl ether vapour. During one week orange plate-shaped crystals suitable for X-ray analysis were obtained. Analysis found: C 46.25, H 4.70, N 12.93%; calculated for C_50_H_60_N_12_Co_3_O_16_Cl: C 46.29, H 4.66, N 12.96%.

### Refinement

Two water molecules are disordered over two sites. The disorder was modelled by placing 5/3 water molecules over four sites with occupancies 2/3 (O15) and 1/3 (O16A, O16B, O16C) as the independent refinement of the occupancy factors of three positions of O16 water molecule gives approximately equal values (0.35, 0.33 and 0.32 for O16A, O16B and O16C, repsectively). The H atoms of O15 and O16 O atoms were omitted. Otherwise, the H_2_O H atoms were located from the difference Fourier map but constrained to ride on their parent atom, with *U*_iso_ = 1.5 *U*_eq_(parent atom). Other H atoms were positioned geometrically and were constrained to ride on their parent atoms, with C—H = 0.95–0.98 Å, and *U*_iso_ = 1.2–1.5 *U*_eq_(parent atom). Attempts to refine any more water molecules did not produce acceptable results. The highest peak is located 2.16 Å from atom O16B and the deepest hole is located 0.57 Å from atom Co3.

### Crystal data

**Table:**

[Co_2_(C_15_H_14_N_4_O_3_)_2_(C_5_H_5_N)_4_(H_2_O)_2_]ClO_4_·3.66H_2_O	*Z* = 2
*M**_r_* = 1232.38	*F*_000_ = 1279
Triclinic, *P*1	*D*_x_ = 1.463 Mg m^−^^3^
Hall symbol: -P 1	Mo *K*α radiation λ = 0.71073 Å
*a* = 10.1128 (2) Å	Cell parameters from 30667 reflections
*b* = 13.9615 (4) Å	θ = 1.0–27.5º
*c* = 20.1840 (6) Å	µ = 0.72 mm^−^^1^
α = 85.969 (2)º	*T* = 120 (2) K
β = 80.844 (2)º	Plate, orange
γ = 84.660 (2)º	0.22 × 0.14 × 0.06 mm
*V* = 2796.79 (13) Å^3^	

### Data collection

**Table:**

Nonius KappaCCD diffractometer	9843 independent reflections
Radiation source: fine-focus sealed tube	7347 reflections with *I* > 2σ(*I*)
Monochromator: horizontally mounted graphite crystal	*R*_int_ = 0.058
Detector resolution: 9 pixels mm^-1^	θ_max_ = 25.0º
*T* = 120(2) K	θ_min_ = 2.4º
φ scans and ω scans with κ offset	*h* = −11→12
Absorption correction: multi-scan(SORTAV; Blessing, 1995)	*k* = −16→16
*T*_min_ = 0.866, *T*_max_ = 0.959	*l* = −24→24
37217 measured reflections	

### Refinement

**Table:**

Refinement on *F*^2^	Secondary atom site location: difference Fourier map
Least-squares matrix: full	Hydrogen site location: inferred from neighbouring sites
*R*[*F*^2^ > 2σ(*F*^2^)] = 0.046	H-atom parameters constrained
*wR*(*F*^2^) = 0.125	*w* = 1/[σ^2^(*F*_o_^2^) + (0.0577*P*)^2^ + 3.0428*P*] where *P* = (*F*_o_^2^ + 2*F*_c_^2^)/3
*S* = 1.04	(Δ/σ)_max_ = 0.002
9843 reflections	Δρ_max_ = 0.89 e Å^−^^3^
763 parameters	Δρ_min_ = −0.89 e Å^−^^3^
Primary atom site location: structure-invariant direct methods	Extinction correction: none

### Special details

**Table:**

Geometry. All e.s.d.'s (except the e.s.d. in the dihedral angle between two l.s. planes) are estimated using the full covariance matrix. The cell e.s.d.'s are taken into account individually in the estimation of e.s.d.'s in distances, angles and torsion angles; correlations between e.s.d.'s in cell parameters are only used when they are defined by crystal symmetry. An approximate (isotropic) treatment of cell e.s.d.'s is used for estimating e.s.d.'s involving l.s. planes.
Refinement. Refinement of *F*^2^ against ALL reflections. The weighted *R*-factor *wR* and goodness of fit *S* are based on *F*^2^, conventional *R*-factors *R* are based on *F*, with *F* set to zero for negative *F*^2^. The threshold expression of *F*^2^ > σ(*F*^2^) is used only for calculating *R*-factors(gt) *etc*. and is not relevant to the choice of reflections for refinement. *R*-factors based on *F*^2^ are statistically about twice as large as those based on *F*, and *R*- factors based on ALL data will be even larger.

### Fractional atomic coordinates and isotropic or equivalent isotropic displacement parameters (Å^2^)

**Table:**

	*x*	*y*	*z*	*U*_iso_*/*U*_eq_	Occ. (<1)
Co1	0.5000	0.5000	0.0000	0.01772 (15)	
Co2	0.21356 (4)	1.07447 (3)	−0.24729 (2)	0.01750 (12)	
Co3	0.5000	0.5000	−0.5000	0.03393 (19)	
Cl1	0.17180 (9)	0.29650 (7)	0.18619 (6)	0.0437 (3)	
O1	0.3981 (2)	0.48640 (16)	0.10222 (11)	0.0238 (5)	
H1O	0.3275	0.5362	0.0988	0.036*	
H1P	0.3550	0.4332	0.1172	0.036*	
O2	0.4367 (2)	0.64311 (14)	−0.00198 (11)	0.0219 (5)	
O3	0.2401 (2)	0.64052 (15)	0.06781 (11)	0.0222 (5)	
O4	0.7502 (3)	1.2930 (2)	−0.31889 (14)	0.0480 (7)	
O5	−0.3956 (2)	0.97963 (18)	−0.18204 (12)	0.0310 (6)	
O6	0.7025 (3)	0.69155 (19)	−0.51207 (15)	0.0457 (7)	
O7	0.4953 (3)	0.64269 (18)	−0.50015 (14)	0.0399 (6)	
O8	0.2835 (3)	0.50375 (19)	−0.47900 (13)	0.0401 (6)	
H8O	0.2240	0.5442	−0.5030	0.060*	
H8P	0.2688	0.4390	−0.4771	0.060*	
O9	0.0962 (4)	0.2458 (3)	0.1503 (3)	0.0960 (14)	
O10	0.3055 (3)	0.2999 (2)	0.15105 (17)	0.0555 (9)	
O11	0.1786 (4)	0.2494 (3)	0.25130 (19)	0.0894 (14)	
O12	0.1115 (4)	0.3924 (2)	0.19205 (19)	0.0725 (10)	
O13	0.8827 (3)	1.4012 (2)	−0.43516 (17)	0.0608 (9)	
H13O	0.8317	1.3753	−0.3979	0.091*	
H13P	0.9545	1.3597	−0.4541	0.091*	
O14	0.9497 (3)	0.7500 (3)	−0.5003 (2)	0.0860 (12)	
H14O	0.9703	0.7543	−0.4563	0.129*	
H14P	0.8572	0.7380	−0.4969	0.129*	
O15	1.0143 (5)	0.7592 (4)	−0.3707 (3)	0.0713 (16)	0.67
O16A	1.1607 (12)	0.5972 (9)	−0.3717 (6)	0.074 (3)	0.33
O16B	1.0770 (14)	0.6548 (10)	−0.3599 (8)	0.100 (5)	0.33
O16C	1.1802 (16)	0.6246 (10)	−0.3162 (7)	0.096 (4)	0.33
N1	0.6795 (3)	0.53403 (19)	0.03527 (14)	0.0230 (6)	
N2	0.1470 (2)	1.04316 (18)	−0.09920 (12)	0.0182 (6)	
H2N	0.0643	1.0357	−0.1146	0.027*	
N3	0.2300 (2)	1.08608 (17)	−0.15337 (13)	0.0178 (6)	
N4	0.3844 (2)	1.12656 (18)	−0.25893 (13)	0.0194 (6)	
N5	0.4711 (2)	1.15209 (18)	−0.31271 (13)	0.0208 (6)	
N6	0.2812 (2)	0.93692 (18)	−0.23800 (13)	0.0201 (6)	
N7	0.1348 (2)	1.20892 (18)	−0.25619 (13)	0.0201 (6)	
N8	−0.0578 (2)	1.02941 (18)	−0.18460 (13)	0.0194 (6)	
N9	0.0359 (2)	1.04090 (18)	−0.23749 (13)	0.0190 (6)	
N10	0.2051 (2)	1.05454 (19)	−0.34023 (13)	0.0201 (6)	
N11	0.3197 (3)	1.05118 (19)	−0.38960 (13)	0.0223 (6)	
H11N	0.3799	1.0952	−0.3785	0.033*	
N12	0.5142 (3)	0.4901 (2)	−0.39281 (16)	0.0387 (8)	
C1	0.7372 (3)	0.4779 (3)	0.08101 (19)	0.0320 (8)	
H1	0.6910	0.4252	0.1026	0.038*	
C2	0.8603 (4)	0.4924 (3)	0.0986 (2)	0.0421 (10)	
H2	0.8966	0.4518	0.1322	0.050*	
C3	0.9287 (4)	0.5675 (3)	0.0659 (2)	0.0440 (11)	
H3	1.0151	0.5779	0.0756	0.053*	
C4	0.8709 (4)	0.6272 (3)	0.0193 (2)	0.0395 (10)	
H4	0.9156	0.6802	−0.0030	0.047*	
C5	0.7469 (3)	0.6086 (2)	0.00551 (19)	0.0286 (8)	
H5	0.7069	0.6501	−0.0264	0.034*	
C6	0.3195 (3)	0.6796 (2)	0.02241 (16)	0.0182 (7)	
C7	0.2778 (3)	0.7762 (2)	−0.00855 (16)	0.0177 (7)	
C8	0.1421 (3)	0.8123 (2)	0.00155 (16)	0.0187 (7)	
H8	0.0776	0.7770	0.0302	0.022*	
C9	0.1020 (3)	0.8986 (2)	−0.02991 (15)	0.0183 (7)	
H9	0.0095	0.9211	−0.0238	0.022*	
C10	0.1953 (3)	0.9536 (2)	−0.07052 (15)	0.0162 (6)	
C11	0.3308 (3)	0.9188 (2)	−0.08003 (15)	0.0184 (7)	
H11	0.3958	0.9557	−0.1069	0.022*	
C12	0.3700 (3)	0.8307 (2)	−0.05040 (16)	0.0186 (7)	
H12	0.4617	0.8065	−0.0587	0.022*	
C13	0.3309 (3)	1.1311 (2)	−0.14138 (16)	0.0188 (7)	
C14	0.3557 (3)	1.1503 (2)	−0.07296 (17)	0.0249 (7)	
H14A	0.2746	1.1405	−0.0404	0.037*	
H14B	0.3785	1.2170	−0.0726	0.037*	
H14C	0.4303	1.1062	−0.0608	0.037*	
C15	0.4178 (3)	1.1587 (2)	−0.20213 (16)	0.0205 (7)	
C16	0.5345 (3)	1.2072 (2)	−0.21908 (17)	0.0241 (7)	
C17	0.6133 (4)	1.2510 (3)	−0.17371 (19)	0.0350 (9)	
H17A	0.6955	1.2737	−0.2001	0.053*	
H17B	0.6370	1.2025	−0.1391	0.053*	
H17C	0.5588	1.3054	−0.1523	0.053*	
C18	0.5634 (3)	1.2011 (2)	−0.28909 (17)	0.0237 (7)	
C19	0.6754 (3)	1.2395 (3)	−0.33711 (19)	0.0320 (8)	
C20	0.6922 (4)	1.2133 (3)	−0.40887 (18)	0.0339 (9)	
H20A	0.6500	1.2654	−0.4355	0.051*	
H20B	0.6493	1.1538	−0.4114	0.051*	
H20C	0.7881	1.2035	−0.4267	0.051*	
C21	0.1994 (3)	0.8673 (2)	−0.21359 (18)	0.0268 (8)	
H21	0.1058	0.8845	−0.2024	0.032*	
C22	0.2465 (4)	0.7726 (3)	−0.2044 (2)	0.0381 (9)	
H22	0.1860	0.7255	−0.1870	0.046*	
C23	0.3820 (4)	0.7463 (3)	−0.2205 (2)	0.0399 (10)	
H23	0.4164	0.6812	−0.2142	0.048*	
C24	0.4662 (3)	0.8162 (2)	−0.24604 (19)	0.0314 (8)	
H24	0.5600	0.8001	−0.2578	0.038*	
C25	0.4128 (3)	0.9100 (2)	−0.25427 (16)	0.0223 (7)	
H25	0.4717	0.9578	−0.2723	0.027*	
C26	0.1629 (3)	1.2650 (2)	−0.31292 (18)	0.0271 (8)	
H26	0.2289	1.2410	−0.3481	0.033*	
C27	0.1002 (4)	1.3552 (3)	−0.32191 (19)	0.0341 (9)	
H27	0.1217	1.3922	−0.3629	0.041*	
C28	0.0055 (4)	1.3921 (3)	−0.27101 (19)	0.0339 (9)	
H28	−0.0385	1.4547	−0.2762	0.041*	
C29	−0.0238 (3)	1.3358 (2)	−0.21226 (18)	0.0283 (8)	
H29	−0.0880	1.3593	−0.1762	0.034*	
C30	0.0415 (3)	1.2449 (2)	−0.20685 (17)	0.0233 (7)	
H30	0.0198	1.2061	−0.1667	0.028*	
C31	−0.2796 (3)	0.9752 (3)	−0.08851 (18)	0.0332 (9)	
H31A	−0.3684	0.9657	−0.0626	0.050*	
H31B	−0.2464	1.0333	−0.0747	0.050*	
H31C	−0.2171	0.9193	−0.0802	0.050*	
C32	−0.2900 (3)	0.9865 (2)	−0.16167 (17)	0.0232 (7)	
C33	−0.1682 (3)	1.0067 (2)	−0.20892 (16)	0.0210 (7)	
C34	−0.1440 (3)	1.0034 (2)	−0.27931 (17)	0.0225 (7)	
C35	−0.2355 (3)	0.9745 (3)	−0.32410 (18)	0.0295 (8)	
H35A	−0.1872	0.9713	−0.3701	0.044*	
H35B	−0.3133	1.0221	−0.3234	0.044*	
H35C	−0.2662	0.9112	−0.3083	0.044*	
C36	−0.0115 (3)	1.0268 (2)	−0.29541 (16)	0.0215 (7)	
C37	0.0898 (3)	1.0350 (2)	−0.35526 (16)	0.0214 (7)	
C38	0.0627 (3)	1.0239 (3)	−0.42464 (17)	0.0297 (8)	
H38A	0.1436	1.0353	−0.4570	0.044*	
H38B	−0.0111	1.0706	−0.4344	0.044*	
H38C	0.0379	0.9584	−0.4282	0.044*	
C39	0.3831 (3)	0.9603 (2)	−0.40649 (16)	0.0224 (7)	
C40	0.3147 (3)	0.8779 (2)	−0.40348 (17)	0.0277 (8)	
H40	0.2228	0.8788	−0.3837	0.033*	
C41	0.3814 (3)	0.7946 (3)	−0.42952 (18)	0.0297 (8)	
H41	0.3341	0.7386	−0.4280	0.036*	
C42	0.5161 (3)	0.7912 (2)	−0.45783 (17)	0.0286 (8)	
C43	0.5868 (3)	0.8706 (2)	−0.45577 (17)	0.0277 (8)	
H43	0.6807	0.8672	−0.4715	0.033*	
C44	0.5215 (3)	0.9551 (2)	−0.43091 (16)	0.0247 (7)	
H44	0.5704	1.0098	−0.4303	0.030*	
C45	0.5781 (4)	0.7027 (3)	−0.49202 (19)	0.0345 (9)	
C46	0.5982 (4)	0.5392 (3)	−0.3651 (2)	0.0433 (10)	
H46	0.6587	0.5775	−0.3940	0.052*	
C47	0.6008 (5)	0.5368 (3)	−0.2967 (2)	0.0493 (11)	
H47	0.6627	0.5717	−0.2793	0.059*	
C48	0.5112 (5)	0.4824 (3)	−0.2540 (2)	0.0484 (11)	
H48	0.5098	0.4799	−0.2067	0.058*	
C49	0.4248 (5)	0.4324 (3)	−0.2814 (2)	0.0499 (11)	
H49	0.3618	0.3951	−0.2535	0.060*	
C50	0.4309 (4)	0.4373 (3)	−0.3511 (2)	0.0459 (10)	
H50	0.3723	0.4009	−0.3697	0.055*	

### Atomic displacement parameters (Å^2^)

**Table:**

	*U*^11^	*U*^22^	*U*^33^	*U*^12^	*U*^13^	*U*^23^
Co1	0.0140 (3)	0.0171 (3)	0.0220 (3)	−0.0012 (2)	−0.0034 (2)	0.0000 (2)
Co2	0.0134 (2)	0.0219 (2)	0.0169 (2)	−0.00310 (16)	−0.00041 (16)	−0.00095 (17)
Co3	0.0423 (4)	0.0287 (4)	0.0295 (4)	0.0038 (3)	−0.0036 (3)	−0.0060 (3)
Cl1	0.0306 (5)	0.0399 (5)	0.0564 (7)	−0.0010 (4)	−0.0013 (4)	0.0120 (5)
O1	0.0211 (11)	0.0249 (12)	0.0248 (13)	−0.0023 (9)	−0.0033 (10)	0.0024 (10)
O2	0.0131 (11)	0.0194 (11)	0.0319 (13)	−0.0020 (9)	0.0004 (9)	0.0005 (10)
O3	0.0187 (11)	0.0218 (11)	0.0248 (13)	−0.0028 (9)	0.0001 (10)	0.0023 (10)
O4	0.0394 (15)	0.070 (2)	0.0379 (17)	−0.0346 (15)	0.0002 (13)	−0.0043 (14)
O5	0.0153 (12)	0.0510 (16)	0.0272 (14)	−0.0082 (10)	−0.0022 (10)	−0.0003 (11)
O6	0.0386 (16)	0.0371 (15)	0.0560 (19)	0.0053 (12)	0.0066 (13)	−0.0077 (13)
O7	0.0453 (16)	0.0295 (14)	0.0451 (17)	0.0017 (12)	−0.0070 (13)	−0.0105 (12)
O8	0.0409 (15)	0.0361 (15)	0.0418 (17)	0.0062 (12)	−0.0070 (12)	−0.0036 (13)
O9	0.074 (3)	0.084 (3)	0.141 (4)	−0.028 (2)	−0.033 (3)	−0.019 (3)
O10	0.0296 (15)	0.0569 (19)	0.072 (2)	−0.0041 (13)	0.0040 (14)	0.0262 (16)
O11	0.067 (2)	0.110 (3)	0.066 (2)	0.031 (2)	0.0190 (19)	0.051 (2)
O12	0.073 (2)	0.055 (2)	0.085 (3)	0.0264 (17)	−0.0149 (19)	0.0010 (18)
O13	0.0453 (18)	0.065 (2)	0.066 (2)	−0.0058 (15)	0.0002 (16)	0.0170 (17)
O14	0.049 (2)	0.083 (3)	0.120 (4)	−0.0022 (18)	−0.002 (2)	0.006 (2)
O15	0.044 (3)	0.087 (4)	0.078 (4)	−0.025 (3)	0.028 (3)	−0.030 (3)
O16A	0.068 (7)	0.083 (8)	0.065 (8)	−0.006 (6)	0.017 (6)	−0.024 (6)
O16B	0.075 (8)	0.080 (9)	0.125 (13)	0.000 (7)	0.043 (9)	−0.010 (8)
O16C	0.130 (12)	0.087 (9)	0.070 (9)	−0.021 (8)	0.007 (8)	−0.026 (7)
N1	0.0185 (14)	0.0263 (15)	0.0247 (16)	0.0007 (11)	−0.0044 (12)	−0.0067 (12)
N2	0.0139 (12)	0.0250 (14)	0.0146 (14)	−0.0015 (10)	−0.0001 (10)	0.0020 (11)
N3	0.0155 (13)	0.0188 (13)	0.0171 (14)	−0.0005 (10)	0.0020 (11)	0.0012 (11)
N4	0.0168 (13)	0.0231 (14)	0.0178 (15)	−0.0018 (11)	−0.0010 (11)	−0.0007 (11)
N5	0.0162 (13)	0.0255 (14)	0.0197 (15)	−0.0051 (11)	0.0014 (11)	−0.0004 (11)
N6	0.0187 (14)	0.0244 (14)	0.0171 (14)	−0.0034 (11)	−0.0016 (11)	−0.0016 (11)
N7	0.0152 (13)	0.0261 (14)	0.0186 (15)	−0.0028 (11)	−0.0008 (11)	−0.0023 (12)
N8	0.0129 (13)	0.0236 (14)	0.0212 (15)	−0.0022 (10)	−0.0009 (11)	−0.0005 (11)
N9	0.0162 (13)	0.0237 (14)	0.0167 (14)	−0.0035 (11)	−0.0002 (11)	−0.0011 (11)
N10	0.0144 (13)	0.0280 (15)	0.0166 (14)	−0.0042 (11)	0.0032 (11)	−0.0021 (11)
N11	0.0183 (13)	0.0276 (15)	0.0196 (15)	−0.0073 (11)	0.0049 (11)	−0.0036 (12)
N12	0.0454 (19)	0.0373 (18)	0.0323 (19)	0.0025 (15)	−0.0037 (15)	−0.0075 (15)
C1	0.0277 (19)	0.037 (2)	0.031 (2)	−0.0026 (16)	−0.0057 (16)	−0.0015 (17)
C2	0.030 (2)	0.061 (3)	0.039 (2)	0.0030 (19)	−0.0191 (18)	−0.011 (2)
C3	0.0213 (19)	0.063 (3)	0.052 (3)	−0.0060 (19)	−0.0067 (18)	−0.029 (2)
C4	0.028 (2)	0.043 (2)	0.050 (3)	−0.0132 (17)	−0.0043 (18)	−0.015 (2)
C5	0.0238 (18)	0.0263 (18)	0.037 (2)	−0.0057 (14)	−0.0039 (15)	−0.0062 (16)
C6	0.0160 (16)	0.0201 (16)	0.0205 (17)	−0.0047 (13)	−0.0060 (13)	−0.0029 (13)
C7	0.0171 (15)	0.0184 (15)	0.0180 (17)	−0.0012 (12)	−0.0027 (13)	−0.0038 (13)
C8	0.0162 (15)	0.0211 (16)	0.0181 (17)	−0.0052 (12)	0.0018 (13)	−0.0016 (13)
C9	0.0110 (14)	0.0252 (17)	0.0186 (17)	−0.0012 (12)	−0.0006 (12)	−0.0040 (13)
C10	0.0152 (15)	0.0179 (15)	0.0149 (16)	−0.0010 (12)	−0.0007 (12)	−0.0021 (12)
C11	0.0154 (15)	0.0218 (16)	0.0171 (17)	−0.0046 (12)	0.0006 (12)	0.0011 (13)
C12	0.0117 (14)	0.0226 (16)	0.0211 (17)	−0.0012 (12)	−0.0009 (13)	−0.0019 (13)
C13	0.0163 (15)	0.0186 (16)	0.0212 (17)	0.0014 (12)	−0.0038 (13)	−0.0002 (13)
C14	0.0247 (17)	0.0293 (18)	0.0213 (18)	−0.0049 (14)	−0.0035 (14)	−0.0018 (14)
C15	0.0189 (16)	0.0203 (16)	0.0226 (18)	−0.0014 (13)	−0.0044 (13)	−0.0001 (13)
C16	0.0207 (17)	0.0258 (17)	0.027 (2)	−0.0060 (13)	−0.0053 (14)	0.0009 (14)
C17	0.035 (2)	0.045 (2)	0.030 (2)	−0.0173 (17)	−0.0081 (16)	−0.0010 (17)
C18	0.0195 (16)	0.0267 (17)	0.0252 (19)	−0.0080 (13)	−0.0002 (14)	−0.0019 (14)
C19	0.0267 (19)	0.036 (2)	0.033 (2)	−0.0087 (16)	−0.0004 (16)	0.0004 (17)
C20	0.031 (2)	0.044 (2)	0.026 (2)	−0.0148 (16)	0.0049 (16)	−0.0007 (17)
C21	0.0212 (17)	0.0273 (18)	0.031 (2)	−0.0053 (14)	0.0022 (14)	−0.0035 (15)
C22	0.032 (2)	0.0259 (19)	0.053 (3)	−0.0068 (16)	0.0058 (18)	0.0016 (18)
C23	0.039 (2)	0.0235 (19)	0.053 (3)	0.0034 (16)	0.0018 (19)	−0.0007 (18)
C24	0.0243 (18)	0.032 (2)	0.035 (2)	0.0044 (15)	0.0008 (15)	−0.0012 (16)
C25	0.0176 (16)	0.0270 (17)	0.0215 (18)	−0.0022 (13)	−0.0002 (13)	−0.0021 (14)
C26	0.0231 (17)	0.0319 (19)	0.0247 (19)	−0.0019 (14)	−0.0001 (14)	0.0013 (15)
C27	0.034 (2)	0.034 (2)	0.030 (2)	0.0004 (16)	0.0003 (16)	0.0104 (16)
C28	0.031 (2)	0.0272 (19)	0.041 (2)	0.0033 (15)	−0.0035 (17)	0.0029 (17)
C29	0.0228 (17)	0.0295 (19)	0.033 (2)	0.0005 (14)	−0.0042 (15)	−0.0095 (16)
C30	0.0209 (16)	0.0292 (18)	0.0202 (18)	−0.0043 (14)	−0.0036 (14)	−0.0005 (14)
C31	0.0183 (17)	0.056 (2)	0.025 (2)	−0.0102 (16)	−0.0002 (14)	0.0055 (17)
C32	0.0196 (17)	0.0236 (17)	0.0258 (19)	−0.0038 (13)	−0.0014 (14)	−0.0003 (14)
C33	0.0163 (16)	0.0235 (17)	0.0221 (18)	−0.0011 (13)	−0.0010 (13)	0.0011 (14)
C34	0.0174 (16)	0.0265 (17)	0.0231 (19)	−0.0021 (13)	−0.0022 (13)	−0.0003 (14)
C35	0.0199 (17)	0.043 (2)	0.027 (2)	−0.0068 (15)	−0.0053 (15)	−0.0021 (16)
C36	0.0181 (16)	0.0259 (17)	0.0208 (18)	−0.0015 (13)	−0.0046 (13)	−0.0005 (14)
C37	0.0197 (16)	0.0244 (17)	0.0196 (18)	−0.0024 (13)	−0.0014 (13)	0.0002 (14)
C38	0.0233 (18)	0.047 (2)	0.0192 (19)	−0.0061 (16)	−0.0030 (14)	−0.0022 (16)
C39	0.0225 (17)	0.0302 (18)	0.0146 (17)	−0.0052 (14)	−0.0002 (13)	−0.0038 (14)
C40	0.0244 (18)	0.035 (2)	0.0232 (19)	−0.0070 (15)	0.0007 (14)	−0.0042 (15)
C41	0.0320 (19)	0.0293 (19)	0.029 (2)	−0.0083 (15)	−0.0026 (15)	−0.0045 (15)
C42	0.0306 (19)	0.0310 (19)	0.0231 (19)	−0.0002 (15)	−0.0009 (15)	−0.0031 (15)
C43	0.0231 (17)	0.036 (2)	0.0227 (19)	−0.0014 (15)	−0.0010 (14)	0.0004 (15)
C44	0.0225 (17)	0.0312 (18)	0.0215 (18)	−0.0077 (14)	−0.0029 (14)	−0.0030 (14)
C45	0.037 (2)	0.032 (2)	0.032 (2)	0.0025 (17)	−0.0007 (17)	−0.0019 (16)
C46	0.046 (2)	0.043 (2)	0.042 (3)	−0.0007 (19)	−0.010 (2)	−0.0085 (19)
C47	0.060 (3)	0.046 (2)	0.045 (3)	0.006 (2)	−0.017 (2)	−0.011 (2)
C48	0.068 (3)	0.044 (2)	0.032 (2)	0.012 (2)	−0.013 (2)	−0.006 (2)
C49	0.058 (3)	0.054 (3)	0.036 (3)	−0.004 (2)	−0.004 (2)	0.001 (2)
C50	0.053 (3)	0.049 (2)	0.036 (2)	−0.006 (2)	−0.007 (2)	−0.003 (2)

### Geometric parameters (Å, °)

**Table:**

Co1—O2^i^	2.040 (2)	C9—C10	1.396 (4)
Co1—O2	2.040 (2)	C9—H9	0.9500
Co1—N1	2.156 (3)	C10—C11	1.398 (4)
Co1—N1^i^	2.156 (3)	C11—C12	1.382 (4)
Co1—O1	2.159 (2)	C11—H11	0.9500
Co1—O1^i^	2.159 (2)	C12—H12	0.9500
Co2—N9	1.876 (2)	C13—C15	1.442 (4)
Co2—N4	1.911 (3)	C13—C14	1.489 (5)
Co2—N10	1.932 (3)	C14—H14A	0.9800
Co2—N3	1.948 (3)	C14—H14B	0.9800
Co2—N7	1.978 (3)	C14—H14C	0.9800
Co2—N6	1.984 (3)	C15—C16	1.399 (4)
Co3—O7^ii^	1.988 (2)	C16—C18	1.404 (5)
Co3—O7	1.988 (2)	C16—C17	1.499 (5)
Co3—O8	2.159 (3)	C17—H17A	0.9800
Co3—O8^ii^	2.159 (3)	C17—H17B	0.9800
Co3—N12	2.185 (3)	C17—H17C	0.9800
Co3—N12^ii^	2.185 (3)	C18—C19	1.483 (5)
Cl1—O9	1.399 (4)	C19—C20	1.498 (5)
Cl1—O12	1.424 (3)	C20—H20A	0.9800
Cl1—O10	1.427 (3)	C20—H20B	0.9800
Cl1—O11	1.436 (3)	C20—H20C	0.9800
O1—H1O	0.9561	C21—C22	1.373 (5)
O1—H1P	0.9058	C21—H21	0.9500
O2—C6	1.281 (4)	C22—C23	1.379 (5)
O3—C6	1.249 (4)	C22—H22	0.9500
O4—C19	1.223 (4)	C23—C24	1.374 (5)
O5—C32	1.218 (4)	C23—H23	0.9500
O6—C45	1.257 (4)	C24—C25	1.378 (5)
O7—C45	1.275 (5)	C24—H24	0.9500
O8—H8O	0.9520	C25—H25	0.9500
O8—H8P	0.9277	C26—C27	1.371 (5)
O13—H13O	0.9137	C26—H26	0.9500
O13—H13P	0.9336	C27—C28	1.381 (5)
O14—H14O	0.9507	C27—H27	0.9500
O14—H14P	0.9563	C28—C29	1.383 (5)
O15—O16B	1.550 (14)	C28—H28	0.9500
O16A—O16B	1.121 (15)	C29—C30	1.381 (5)
O16A—O16C	1.260 (16)	C29—H29	0.9500
O16B—O16C	1.48 (2)	C30—H30	0.9500
N1—C1	1.338 (4)	C31—C32	1.493 (5)
N1—C5	1.350 (4)	C31—H31A	0.9800
N2—N3	1.403 (3)	C31—H31B	0.9800
N2—C10	1.419 (4)	C31—H31C	0.9800
N2—H2N	0.9539	C32—C33	1.469 (4)
N3—C13	1.309 (4)	C33—C34	1.406 (5)
N4—N5	1.332 (4)	C34—C36	1.392 (4)
N4—C15	1.361 (4)	C34—C35	1.491 (5)
N5—C18	1.369 (4)	C35—H35A	0.9800
N6—C25	1.344 (4)	C35—H35B	0.9800
N6—C21	1.350 (4)	C35—H35C	0.9800
N7—C26	1.348 (4)	C36—C37	1.460 (4)
N7—C30	1.348 (4)	C37—C38	1.490 (5)
N8—N9	1.321 (4)	C38—H38A	0.9800
N8—C33	1.360 (4)	C38—H38B	0.9800
N9—C36	1.365 (4)	C38—H38C	0.9800
N10—C37	1.307 (4)	C39—C40	1.390 (5)
N10—N11	1.402 (3)	C39—C44	1.405 (4)
N11—C39	1.406 (4)	C40—C41	1.382 (5)
N11—H11N	0.9658	C40—H40	0.9500
N12—C50	1.329 (5)	C41—C42	1.389 (5)
N12—C46	1.349 (5)	C41—H41	0.9500
C1—C2	1.383 (5)	C42—C43	1.380 (5)
C1—H1	0.9500	C42—C45	1.494 (5)
C2—C3	1.381 (6)	C43—C44	1.383 (5)
C2—H2	0.9500	C43—H43	0.9500
C3—C4	1.378 (6)	C44—H44	0.9500
C3—H3	0.9500	C46—C47	1.383 (6)
C4—C5	1.378 (5)	C46—H46	0.9500
C4—H4	0.9500	C47—C48	1.389 (6)
C5—H5	0.9500	C47—H47	0.9500
C6—C7	1.499 (4)	C48—C49	1.372 (6)
C7—C12	1.397 (4)	C48—H48	0.9500
C7—C8	1.405 (4)	C49—C50	1.395 (6)
C8—C9	1.380 (4)	C49—H49	0.9500
C8—H8	0.9500	C50—H50	0.9500
			
O2^i^—Co1—O2	180.00 (13)	C7—C12—H12	119.2
O2^i^—Co1—N1	91.07 (9)	N3—C13—C15	112.3 (3)
O2—Co1—N1	88.93 (9)	N3—C13—C14	124.3 (3)
O2^i^—Co1—N1^i^	88.93 (9)	C15—C13—C14	123.3 (3)
O2—Co1—N1^i^	91.07 (9)	C13—C14—H14A	109.5
N1—Co1—N1^i^	180.0	C13—C14—H14B	109.5
O2^i^—Co1—O1	91.36 (8)	H14A—C14—H14B	109.5
O2—Co1—O1	88.64 (8)	C13—C14—H14C	109.5
N1—Co1—O1	90.39 (9)	H14A—C14—H14C	109.5
N1^i^—Co1—O1	89.61 (9)	H14B—C14—H14C	109.5
O2^i^—Co1—O1^i^	88.64 (8)	N4—C15—C16	109.0 (3)
O2—Co1—O1^i^	91.36 (8)	N4—C15—C13	114.1 (3)
N1—Co1—O1^i^	89.61 (9)	C16—C15—C13	136.8 (3)
N1^i^—Co1—O1^i^	90.39 (9)	C15—C16—C18	102.8 (3)
O1—Co1—O1^i^	180.000 (1)	C15—C16—C17	128.9 (3)
N9—Co2—N4	172.10 (11)	C18—C16—C17	128.3 (3)
N9—Co2—N10	81.20 (11)	C16—C17—H17A	109.5
N4—Co2—N10	98.79 (11)	C16—C17—H17B	109.5
N9—Co2—N3	99.94 (11)	H17A—C17—H17B	109.5
N4—Co2—N3	80.63 (11)	C16—C17—H17C	109.5
N10—Co2—N3	175.98 (10)	H17A—C17—H17C	109.5
N9—Co2—N7	85.42 (11)	H17B—C17—H17C	109.5
N4—Co2—N7	86.69 (10)	N5—C18—C16	111.6 (3)
N10—Co2—N7	91.73 (11)	N5—C18—C19	119.3 (3)
N3—Co2—N7	92.21 (10)	C16—C18—C19	129.1 (3)
N9—Co2—N6	91.00 (11)	O4—C19—C18	120.8 (3)
N4—Co2—N6	96.89 (10)	O4—C19—C20	120.9 (3)
N10—Co2—N6	87.54 (11)	C18—C19—C20	118.3 (3)
N3—Co2—N6	88.58 (10)	C19—C20—H20A	109.5
N7—Co2—N6	176.42 (10)	C19—C20—H20B	109.5
O7^ii^—Co3—O7	180.0	H20A—C20—H20B	109.5
O7^ii^—Co3—O8	88.11 (10)	C19—C20—H20C	109.5
O7—Co3—O8	91.89 (10)	H20A—C20—H20C	109.5
O7^ii^—Co3—O8^ii^	91.89 (10)	H20B—C20—H20C	109.5
O7—Co3—O8^ii^	88.11 (10)	N6—C21—C22	122.5 (3)
O8—Co3—O8^ii^	180.0	N6—C21—H21	118.7
O7^ii^—Co3—N12	90.33 (12)	C22—C21—H21	118.7
O7—Co3—N12	89.67 (12)	C21—C22—C23	119.6 (3)
O8—Co3—N12	91.42 (11)	C21—C22—H22	120.2
O8^ii^—Co3—N12	88.58 (11)	C23—C22—H22	120.2
O7^ii^—Co3—N12^ii^	89.67 (12)	C24—C23—C22	118.6 (3)
O7—Co3—N12^ii^	90.33 (12)	C24—C23—H23	120.7
O8—Co3—N12^ii^	88.58 (11)	C22—C23—H23	120.7
O8^ii^—Co3—N12^ii^	91.42 (11)	C23—C24—C25	119.1 (3)
N12—Co3—N12^ii^	180.0	C23—C24—H24	120.4
O9—Cl1—O12	108.7 (2)	C25—C24—H24	120.4
O9—Cl1—O10	110.0 (3)	N6—C25—C24	123.0 (3)
O12—Cl1—O10	108.7 (2)	N6—C25—H25	118.5
O9—Cl1—O11	110.2 (3)	C24—C25—H25	118.5
O12—Cl1—O11	110.9 (2)	N7—C26—C27	122.6 (3)
O10—Cl1—O11	108.22 (19)	N7—C26—H26	118.7
Co1—O1—H1O	97.9	C27—C26—H26	118.7
Co1—O1—H1P	120.3	C26—C27—C28	119.6 (3)
H1O—O1—H1P	104.5	C26—C27—H27	120.2
C6—O2—Co1	125.86 (19)	C28—C27—H27	120.2
C45—O7—Co3	135.3 (2)	C27—C28—C29	118.5 (3)
Co3—O8—H8O	124.6	C27—C28—H28	120.8
Co3—O8—H8P	102.4	C29—C28—H28	120.8
H8O—O8—H8P	113.8	C30—C29—C28	119.0 (3)
H13O—O13—H13P	114.4	C30—C29—H29	120.5
H14O—O14—H14P	108.7	C28—C29—H29	120.5
O16B—O16A—O16C	76.9 (12)	N7—C30—C29	122.7 (3)
O16A—O16B—O16C	55.8 (10)	N7—C30—H30	118.6
O16A—O16B—O15	150.5 (12)	C29—C30—H30	118.6
O16C—O16B—O15	125.4 (13)	C32—C31—H31A	109.5
O16A—O16C—O16B	47.3 (9)	C32—C31—H31B	109.5
C1—N1—C5	117.0 (3)	H31A—C31—H31B	109.5
C1—N1—Co1	122.8 (2)	C32—C31—H31C	109.5
C5—N1—Co1	119.8 (2)	H31A—C31—H31C	109.5
N3—N2—C10	117.8 (2)	H31B—C31—H31C	109.5
N3—N2—H2N	106.5	O5—C32—C33	120.6 (3)
C10—N2—H2N	109.1	O5—C32—C31	121.6 (3)
C13—N3—N2	119.0 (3)	C33—C32—C31	117.8 (3)
C13—N3—Co2	116.9 (2)	N8—C33—C34	111.6 (3)
N2—N3—Co2	123.84 (19)	N8—C33—C32	119.3 (3)
N5—N4—C15	110.8 (2)	C34—C33—C32	129.1 (3)
N5—N4—Co2	133.5 (2)	C36—C34—C33	102.7 (3)
C15—N4—Co2	114.7 (2)	C36—C34—C35	129.3 (3)
N4—N5—C18	105.8 (3)	C33—C34—C35	127.9 (3)
C25—N6—C21	117.3 (3)	C34—C35—H35A	109.5
C25—N6—Co2	120.5 (2)	C34—C35—H35B	109.5
C21—N6—Co2	122.2 (2)	H35A—C35—H35B	109.5
C26—N7—C30	117.5 (3)	C34—C35—H35C	109.5
C26—N7—Co2	122.1 (2)	H35A—C35—H35C	109.5
C30—N7—Co2	120.2 (2)	H35B—C35—H35C	109.5
N9—N8—C33	106.0 (3)	N9—C36—C34	108.8 (3)
N8—N9—C36	110.9 (2)	N9—C36—C37	113.2 (3)
N8—N9—Co2	132.8 (2)	C34—C36—C37	137.9 (3)
C36—N9—Co2	116.2 (2)	N10—C37—C36	111.6 (3)
C37—N10—N11	119.8 (3)	N10—C37—C38	124.9 (3)
C37—N10—Co2	117.6 (2)	C36—C37—C38	123.5 (3)
N11—N10—Co2	122.29 (19)	C37—C38—H38A	109.5
N10—N11—C39	118.1 (2)	C37—C38—H38B	109.5
N10—N11—H11N	108.0	H38A—C38—H38B	109.5
C39—N11—H11N	113.7	C37—C38—H38C	109.5
C50—N12—C46	117.0 (4)	H38A—C38—H38C	109.5
C50—N12—Co3	119.1 (3)	H38B—C38—H38C	109.5
C46—N12—Co3	123.8 (3)	C40—C39—C44	119.2 (3)
N1—C1—C2	123.6 (4)	C40—C39—N11	123.4 (3)
N1—C1—H1	118.2	C44—C39—N11	117.4 (3)
C2—C1—H1	118.2	C41—C40—C39	119.4 (3)
C3—C2—C1	118.1 (4)	C41—C40—H40	120.3
C3—C2—H2	120.9	C39—C40—H40	120.3
C1—C2—H2	120.9	C40—C41—C42	121.3 (3)
C4—C3—C2	119.5 (3)	C40—C41—H41	119.3
C4—C3—H3	120.3	C42—C41—H41	119.3
C2—C3—H3	120.3	C43—C42—C41	119.1 (3)
C5—C4—C3	118.6 (4)	C43—C42—C45	122.2 (3)
C5—C4—H4	120.7	C41—C42—C45	118.6 (3)
C3—C4—H4	120.7	C42—C43—C44	120.3 (3)
N1—C5—C4	123.1 (3)	C42—C43—H43	119.8
N1—C5—H5	118.4	C44—C43—H43	119.8
C4—C5—H5	118.4	C43—C44—C39	120.3 (3)
O3—C6—O2	125.3 (3)	C43—C44—H44	119.9
O3—C6—C7	119.4 (3)	C39—C44—H44	119.9
O2—C6—C7	115.3 (3)	O6—C45—O7	124.0 (3)
C12—C7—C8	118.2 (3)	O6—C45—C42	121.1 (3)
C12—C7—C6	121.6 (3)	O7—C45—C42	114.9 (3)
C8—C7—C6	120.2 (3)	N12—C46—C47	123.3 (4)
C9—C8—C7	120.4 (3)	N12—C46—H46	118.3
C9—C8—H8	119.8	C47—C46—H46	118.3
C7—C8—H8	119.8	C46—C47—C48	118.7 (4)
C8—C9—C10	121.0 (3)	C46—C47—H47	120.7
C8—C9—H9	119.5	C48—C47—H47	120.7
C10—C9—H9	119.5	C49—C48—C47	118.6 (4)
C9—C10—C11	118.9 (3)	C49—C48—H48	120.7
C9—C10—N2	117.7 (2)	C47—C48—H48	120.7
C11—C10—N2	123.4 (3)	C48—C49—C50	119.0 (4)
C12—C11—C10	120.0 (3)	C48—C49—H49	120.5
C12—C11—H11	120.0	C50—C49—H49	120.5
C10—C11—H11	120.0	N12—C50—C49	123.4 (4)
C11—C12—C7	121.5 (3)	N12—C50—H50	118.3
C11—C12—H12	119.2	C49—C50—H50	118.3
			
N1—Co1—O2—C6	−132.2 (3)	C9—C10—C11—C12	1.1 (5)
N1^i^—Co1—O2—C6	47.8 (3)	N2—C10—C11—C12	−180.0 (3)
O1—Co1—O2—C6	−41.8 (3)	C10—C11—C12—C7	−2.5 (5)
O1^i^—Co1—O2—C6	138.2 (3)	C8—C7—C12—C11	1.6 (5)
O8—Co3—O7—C45	158.4 (4)	C6—C7—C12—C11	178.6 (3)
O8^ii^—Co3—O7—C45	−21.6 (4)	N2—N3—C13—C15	171.4 (2)
N12—Co3—O7—C45	67.0 (4)	Co2—N3—C13—C15	−3.8 (3)
N12^ii^—Co3—O7—C45	−113.0 (4)	N2—N3—C13—C14	−7.5 (4)
O2^i^—Co1—N1—C1	−37.8 (3)	Co2—N3—C13—C14	177.3 (2)
O2—Co1—N1—C1	142.2 (3)	N5—N4—C15—C16	−0.9 (3)
O1—Co1—N1—C1	53.6 (3)	Co2—N4—C15—C16	−171.6 (2)
O1^i^—Co1—N1—C1	−126.4 (3)	N5—N4—C15—C13	−178.1 (2)
O2^i^—Co1—N1—C5	134.5 (2)	Co2—N4—C15—C13	11.3 (3)
O2—Co1—N1—C5	−45.5 (2)	N3—C13—C15—N4	−4.8 (4)
O1—Co1—N1—C5	−134.1 (2)	C14—C13—C15—N4	174.1 (3)
O1^i^—Co1—N1—C5	45.9 (2)	N3—C13—C15—C16	179.1 (3)
C10—N2—N3—C13	−77.9 (3)	C14—C13—C15—C16	−1.9 (6)
C10—N2—N3—Co2	97.0 (3)	N4—C15—C16—C18	0.7 (3)
N9—Co2—N3—C13	−164.1 (2)	C13—C15—C16—C18	176.9 (3)
N4—Co2—N3—C13	7.9 (2)	N4—C15—C16—C17	−176.8 (3)
N7—Co2—N3—C13	−78.4 (2)	C13—C15—C16—C17	−0.7 (6)
N6—Co2—N3—C13	105.1 (2)	N4—N5—C18—C16	−0.3 (3)
N9—Co2—N3—N2	20.8 (2)	N4—N5—C18—C19	179.8 (3)
N4—Co2—N3—N2	−167.1 (2)	C15—C16—C18—N5	−0.3 (4)
N7—Co2—N3—N2	106.6 (2)	C17—C16—C18—N5	177.3 (3)
N6—Co2—N3—N2	−69.9 (2)	C15—C16—C18—C19	179.7 (3)
N10—Co2—N4—N5	5.8 (3)	C17—C16—C18—C19	−2.8 (6)
N3—Co2—N4—N5	−178.2 (3)	N5—C18—C19—O4	171.4 (3)
N7—Co2—N4—N5	−85.4 (3)	C16—C18—C19—O4	−8.5 (6)
N6—Co2—N4—N5	94.4 (3)	N5—C18—C19—C20	−6.9 (5)
N10—Co2—N4—C15	173.7 (2)	C16—C18—C19—C20	173.2 (3)
N3—Co2—N4—C15	−10.3 (2)	C25—N6—C21—C22	0.9 (5)
N7—Co2—N4—C15	82.5 (2)	Co2—N6—C21—C22	−177.2 (3)
N6—Co2—N4—C15	−97.7 (2)	N6—C21—C22—C23	−0.1 (6)
C15—N4—N5—C18	0.7 (3)	C21—C22—C23—C24	−0.5 (6)
Co2—N4—N5—C18	169.0 (2)	C22—C23—C24—C25	0.3 (6)
N9—Co2—N6—C25	166.5 (2)	C21—N6—C25—C24	−1.1 (5)
N4—Co2—N6—C25	−13.2 (3)	Co2—N6—C25—C24	177.0 (3)
N10—Co2—N6—C25	85.3 (2)	C23—C24—C25—N6	0.5 (5)
N3—Co2—N6—C25	−93.6 (2)	C30—N7—C26—C27	−0.5 (5)
N9—Co2—N6—C21	−15.5 (3)	Co2—N7—C26—C27	174.2 (3)
N4—Co2—N6—C21	164.8 (3)	N7—C26—C27—C28	1.0 (6)
N10—Co2—N6—C21	−96.7 (3)	C26—C27—C28—C29	−0.5 (6)
N3—Co2—N6—C21	84.4 (3)	C27—C28—C29—C30	−0.5 (5)
N9—Co2—N7—C26	−117.0 (3)	C26—N7—C30—C29	−0.6 (5)
N4—Co2—N7—C26	62.7 (3)	Co2—N7—C30—C29	−175.3 (2)
N10—Co2—N7—C26	−36.0 (3)	C28—C29—C30—N7	1.1 (5)
N3—Co2—N7—C26	143.2 (3)	N9—N8—C33—C34	0.1 (3)
N9—Co2—N7—C30	57.5 (2)	N9—N8—C33—C32	178.3 (3)
N4—Co2—N7—C30	−122.8 (2)	O5—C32—C33—N8	169.4 (3)
N10—Co2—N7—C30	138.5 (2)	C31—C32—C33—N8	−10.4 (4)
N3—Co2—N7—C30	−42.3 (2)	O5—C32—C33—C34	−12.7 (5)
C33—N8—N9—C36	0.4 (3)	C31—C32—C33—C34	167.4 (3)
C33—N8—N9—Co2	178.8 (2)	N8—C33—C34—C36	−0.6 (3)
N10—Co2—N9—N8	177.6 (3)	C32—C33—C34—C36	−178.5 (3)
N3—Co2—N9—N8	1.5 (3)	N8—C33—C34—C35	175.3 (3)
N7—Co2—N9—N8	−90.0 (3)	C32—C33—C34—C35	−2.7 (5)
N6—Co2—N9—N8	90.2 (3)	N8—N9—C36—C34	−0.8 (3)
N10—Co2—N9—C36	−4.1 (2)	Co2—N9—C36—C34	−179.5 (2)
N3—Co2—N9—C36	179.8 (2)	N8—N9—C36—C37	−177.8 (2)
N7—Co2—N9—C36	88.4 (2)	Co2—N9—C36—C37	3.5 (3)
N6—Co2—N9—C36	−91.4 (2)	C33—C34—C36—N9	0.8 (3)
N9—Co2—N10—C37	4.1 (2)	C35—C34—C36—N9	−175.0 (3)
N4—Co2—N10—C37	−167.9 (2)	C33—C34—C36—C37	176.6 (4)
N7—Co2—N10—C37	−81.0 (2)	C35—C34—C36—C37	0.9 (6)
N6—Co2—N10—C37	95.5 (2)	N11—N10—C37—C36	170.7 (3)
N9—Co2—N10—N11	−169.6 (2)	Co2—N10—C37—C36	−3.2 (3)
N4—Co2—N10—N11	18.4 (2)	N11—N10—C37—C38	−10.3 (5)
N7—Co2—N10—N11	105.3 (2)	Co2—N10—C37—C38	175.8 (2)
N6—Co2—N10—N11	−78.2 (2)	N9—C36—C37—N10	−0.2 (4)
C37—N10—N11—C39	−76.9 (4)	C34—C36—C37—N10	−175.9 (4)
Co2—N10—N11—C39	96.7 (3)	N9—C36—C37—C38	−179.2 (3)
O7^ii^—Co3—N12—C50	−50.1 (3)	C34—C36—C37—C38	5.1 (6)
O7—Co3—N12—C50	129.9 (3)	N10—N11—C39—C40	30.9 (5)
O8—Co3—N12—C50	38.0 (3)	N10—N11—C39—C44	−152.5 (3)
O8^ii^—Co3—N12—C50	−142.0 (3)	C44—C39—C40—C41	−5.2 (5)
O7^ii^—Co3—N12—C46	133.9 (3)	N11—C39—C40—C41	171.3 (3)
O7—Co3—N12—C46	−46.1 (3)	C39—C40—C41—C42	0.9 (5)
O8—Co3—N12—C46	−138.0 (3)	C40—C41—C42—C43	4.4 (5)
O8^ii^—Co3—N12—C46	42.0 (3)	C40—C41—C42—C45	−173.1 (3)
Co1—N1—C1—C2	172.3 (3)	C41—C42—C43—C44	−5.4 (5)
N1—C1—C2—C3	−1.6 (6)	C45—C42—C43—C44	172.1 (3)
C1—C2—C3—C4	2.4 (6)	C42—C43—C44—C39	1.1 (5)
C2—C3—C4—C5	−1.4 (6)	C40—C39—C44—C43	4.3 (5)
C1—N1—C5—C4	1.3 (5)	N11—C39—C44—C43	−172.4 (3)
Co1—N1—C5—C4	−171.4 (3)	Co3—O7—C45—O6	35.7 (6)
C3—C4—C5—N1	−0.5 (5)	Co3—O7—C45—C42	−145.9 (3)
Co1—O2—C6—O3	23.2 (4)	C43—C42—C45—O6	10.3 (6)
Co1—O2—C6—C7	−155.6 (2)	C41—C42—C45—O6	−172.3 (3)
O3—C6—C7—C12	168.3 (3)	C43—C42—C45—O7	−168.2 (3)
O2—C6—C7—C12	−12.8 (4)	C41—C42—C45—O7	9.3 (5)
O3—C6—C7—C8	−14.7 (4)	C50—N12—C46—C47	0.0 (6)
O2—C6—C7—C8	164.1 (3)	Co3—N12—C46—C47	176.1 (3)
C12—C7—C8—C9	0.7 (5)	N12—C46—C47—C48	−1.1 (6)
C6—C7—C8—C9	−176.3 (3)	C46—C47—C48—C49	0.7 (6)
C7—C8—C9—C10	−2.1 (5)	C47—C48—C49—C50	0.6 (6)
C8—C9—C10—C11	1.2 (5)	C46—N12—C50—C49	1.4 (6)
C8—C9—C10—N2	−177.8 (3)	Co3—N12—C50—C49	−174.8 (3)
N3—N2—C10—C9	−163.1 (3)	C48—C49—C50—N12	−1.8 (6)
N3—N2—C10—C11	17.9 (4)		

Symmetry codes: (i) −*x*+1, −*y*+1, −*z*; (ii) −*x*+1, −*y*+1, −*z*−1.

### Hydrogen-bond geometry (Å, °)

**Table:**

*D*—H···*A*	*D*—H	H···*A*	*D*···*A*	*D*—H···*A*
O1—H1O···O3	0.96	1.76	2.677 (3)	159
O1—H1P···O10	0.91	2.02	2.903 (4)	166
O1—H1P···Cl1	0.91	2.91	3.795 (2)	166
O8—H8O···O13^iii^	0.95	1.86	2.789 (4)	166
O8—H8P···O6^ii^	0.93	1.84	2.734 (4)	161
O13—H13O···O4	0.91	2.01	2.909 (4)	167
O13—H13P···O14^iv^	0.93	1.94	2.835 (5)	160
O14—H14O···O15	0.95	1.86	2.809 (7)	178
O14—H14P···O6	0.96	1.83	2.751 (5)	162
N2—H2N···N8	0.95	2.03	2.924 (4)	155
N2—H2N···N9	0.95	2.54	3.173 (4)	124
N11—H11N···N5	0.97	1.98	2.864 (4)	151
N11—H11N···N4	0.97	2.49	3.092 (4)	120

Symmetry codes: (iii) −*x*+1, −*y*+2, −*z*−1; (ii) −*x*+1, −*y*+1, −*z*−1; (iv) −*x*+2, −*y*+2, −*z*−1.

## References

[bb1] Blessing, R. H. (1995). *Acta Cryst.* A**51**, 33–38. 10.1107/s0108767394005726 7702794

[bb2] Brandenburg, K. (2006). *DIAMOND* Version 3.1d. Crystal Impact GbR, Bonn, Germany.

[bb3] Bruker (2004). *COLLECT* Bruker AXS Inc., Madison, Wisconsin, USA.

[bb4] Burla, M. C., Caliandro, R., Camalli, M., Carrozzini, B., Cascarano, G. L., De Caro, L., Giacovazzo, C., Polidori, G. & Spagna, R. (2005). *J. Appl. Cryst.* **38**, 381–388.

[bb5] Dalai, S., Mukherjee, P. S., Drew, M. G. B., Lu, T.-H. & Chaudhuri, N. R. (2002). *Inorg. Chim. Acta*, **335**, 85–90.

[bb6] Eisenwiener, A., Neuburger, M. & Kaden, T. A. (2007). *Dalton Trans.* pp. 218–233. 10.1039/b612948j 17180190

[bb7] James, S. (2003). *Chem. Soc. Rev.* **32**, 276–288.

[bb8] Li, X.-H. & Xiao, H.-P. (2004). *Acta Cryst.* E**60**, m898–m900.

[bb9] Min, D., Yoon, S. S. & Lee, S. W. (2002). *Inorg. Chem. Commun.* **5**, 143–146.

[bb10] Mukherjee, R. (2000). *Coord. Chem. Rev.* **203**, 151–218.

[bb11] Otwinowski, Z. & Minor, W. (1997). *Methods in Enzymology*, Vol. 276, *Macromolecular Crystallography*, Part A, edited by C. W. Carter Jr & R. M. Sweet, pp. 307–326. New York: Academic Press.

[bb12] Sato, S., Fukuda, T., Ishii, K., Nakano, Y. & Fujii, Y. (1999). *Acta Cryst.* C**55**, 1466–1470.

[bb13] Sheldrick, G. M. (2008). *Acta Cryst.* A**64**, 112–122. 10.1107/S0108767307043930 18156677

[bb14] Takahashi, P. M., Melo, L. P., Frem, R. C. G., Netto, A. V. G., Mauro, A. E., Santos, R. H. A. & Ferreira, J. G. (2006). *J. Mol. Struct.* **783**, 161–167.

[bb15] Xiao, H.-P., Wang, J.-G., Li, X.-H., Hu, M.-L. & Zhang, W.-B. (2005). *Acta Cryst.* E**61**, m257–m259.

[bb16] Yin, G., Zhang, Y., Li, B. & Zhang, Y. (2007). *J. Mol. Struct.* **837**, 263–268.

[bb17] Zhu, Z.-B., Gao, S., Liu, J.-W., Huo, L.-H. & Zhao, H. (2004). *Acta Cryst.* E**60**, m808–m810.

